# TRICALS risk score augments but cannot replace neurofilament as a prognostic biomarker in ALS

**DOI:** 10.1007/s00415-026-14040-4

**Published:** 2026-10-06

**Authors:** Ahmad Al Khleifat, Andrea Malaspina, Janine Kirby, Timothy Tree, Pamela J. Shaw, P. Nigel Leigh, Ammar Al-Chalabi

**Affiliations:** 1 Department of Basic and Clinical Neuroscience, Institute of Psychiatry, Psychology and Neuroscience, King’s College London, 16 De Crespigny Park, London, SE5 8AF UK; 2 School of Medicine, The University of Jordan, Amman, Jordan; 3 Perron Institute for Neurological and Translational Science, Nedlands, Australia; 4 UCL Queen Square MND Care and Research Centre, Institute of Neurology, University College London, London, UK; 5 Centre of Neuroscience and Trauma, Blizard Institute, Queen Mary University London, London, UK; 6 Sheffield Institute for Translational Neuroscience, School of Medicine and Population Health, University of Sheffield, Sheffield, UK; 7 Department of Immunobiology, Faculty of Life Sciences and Medicine, King’s College London, London, UK; 8 NIHR Sheffield Biomedical Research Centre, University of Sheffield and Sheffield Teaching Hospitals NHS Foundation Trust, Sheffield, UK; 9 Department of Neuroscience, Brighton and Sussex Medical School, Falmer, Brighton, BN1 9PX UK; 10 Department of Clinical Neurosciences, King’s College Hospital, London, SE5 9RS UK

**Keywords:** Amyotrophic lateral sclerosis, ALS, Biomarker, Clinical trials

## Abstract

**Background:**

Amyotrophic lateral sclerosis (ALS) is a neurodegenerative disorder with substantial clinical heterogeneity, complicating the design and interpretation of clinical trials. Two strategies have been proposed to address this variability: phenotype-based prognostic modelling and biomarker-based stratification using neurofilaments. We aimed to determine whether a widely used phenotype-based method, the TRICALS risk score, could substitute for neurofilament biomarkers in a clinical trial.

**Methods:**

We used data from the MIROCALS phase 2b randomised controlled trial of low-dose interleukin-2 in ALS (*n* = 220). The TRICALS risk score was calculated for each participant. The published biomarker model includes CSF phosphorylated neurofilament heavy chain (pNFH) and a pNFH × treatment interaction. We analysed the effects of replacing CSF pNFH with the TRICALS risk score and of adding TRICALS score as an additional covariate to the original model. Model performance was assessed using likelihood ratio χ^2^ statistics and − 2 log likelihood (− 2LL). The primary outcome was survival.

**Results:**

TRICALS risk score was moderately correlated with CSF pNFH (*r* = 0.357), explaining 12.7% of shared variance. Substituting CSF pNFH with TRICALS score reduced model performance (− 2LL 928.25 vs 793.36). The TRICALS × treatment interaction was statistically significant (hazard ratio [HR] 0.72, 95% CI 0.54–0.96 *p* = 0.025), but contributed modestly to model fit (Δχ^2^ = 4.76), compared with the pNFH × treatment interaction (Δχ^2^ = 14.24). Incorporating the pNFH × treatment interaction significantly improved model fit and identified a treatment effect (HR 0.28, 95% CI 0.12–0.63; *p* = 0.002).

**Conclusions:**

The TRICALS risk score does not substitute for CSF neurofilament biomarkers as a prognostic or stratification variable but provides complementary information.

**Supplementary Information:**

The online version contains supplementary material available at https://doi.org/10.1007/s00415-026-14040-4.

## Introduction

Amyotrophic lateral sclerosis (ALS) is a progressive neurodegenerative disease characterised by marked clinical and biological heterogeneity [1]. This heterogeneity poses major challenges for clinical trial design, statistical power, and interpretation of treatment effects [2]. Contemporary ALS trials frequently adjust for multiple baseline covariates, including age, ALS Functional Rating Scale (ALSFRS-R), and site of onset [2, 3]. Although this approach aims to improve precision, it introduces complexity, increases the risk of model instability, and complicates replication across cohorts and platforms. Composite prognostic models offer an alternative strategy by integrating correlated clinical variables into a single measure that captures baseline disease severity more efficiently and may provide a practical anchor for biomarker-informed survival modelling in clinical trials. 

The TRICALS survival prediction model, derived from more than 10,000 individuals across international cohorts, combines variables including age, diagnostic delay, ALSFRS-R, site of onset, and respiratory status into a validated prognostic index that robustly predicts survival [4]. Its use in clinical trials has the potential to streamline covariate adjustment and improve statistical interpretability. However, it remains unclear whether such phenotype-based models and molecular biomarkers represent the same underlying biological processes and could, therefore, be used interchangeably, or whether they capture distinct dimensions of disease biology that provide complementary information for survival modelling and treatment stratification [5, 6].

Low-dose interleukin-2 (IL-2_LD_) is an immunomodulatory therapy that enhances regulatory T-cell function, studied in early-phase ALS trials. In a randomised placebo-controlled trial of IL-2_LD_, MIROCALS, survival was modelled using a multivariable Cox framework including ALSFRS-R, age, regulatory T-cell frequency, plasma CCL2, Cerebrospinal fluid (CSF) phosphorylated neurofilament heavy chain (pNFH), and a pNFH × treatment interaction. This analysis demonstrated that CSF pNFH is both a strong prognostic marker of disease progression and a modifier of IL-2_LD_ treatment effect, suggesting that neuroaxonal injury burden plays a central role in treatment-response heterogeneity [7].

In this study, we used the MIROCALS trial dataset to address two prespecified questions of direct relevance to ALS trial design. First, can a clinical phenotype model (TRICALS score) substitute for a molecular biomarker (CSF pNFH) in survival modelling and treatment stratification? Second, does clinical phenotype add independent biological information when incorporated into a biomarker-based framework? By performing substitution and augmentation analyses within a unified survival modelling strategy, we aimed to determine whether clinical and molecular predictors of survival are interchangeable or complementary, and to define a biologically coherent predictive framework for biomarker-guided ALS clinical trials.

## Methods

### Study design and participants

This analysis used data from MIROCALS, a multicentre, randomised, double-blind, placebo-controlled Phase 2b trial evaluating IL-2_LD_ in ALS. The participants were randomly allocated 1:1 to IL-2_LD_ or placebo, stratified by clinical site and baseline disease severity. The present study included all randomised participants. All had complete baseline clinical data and biomarker measurements required for the analyses. Survival time was defined as time from randomisation to death, tracheostomy, or permanent assisted ventilation, consistent with the original trial’s primary endpoint. Participants without an event were censored at last known follow-up [7].

### Clinical and biomarker variables

Continuous variables were mean-centred and standardised for stability in regression. Categorical variables were binary coded. Baseline demographic and clinical variables included age, sex (female = 1), site of onset (bulbar = 1), ALS Functional Rating Scale–Revised (ALSFRS-R), and disease duration (months from symptom onset to baseline).

### Biomarker measurements

Biomarker methods are previously published, but in brief, blood and cerebrospinal fluid (CSF) were collected at baseline and stored at − 80 °C [7]. All assays were performed blinded to treatment allocation, in ≤ 2 batches, with internal quality-control samples (coefficient of variation < 10%).

- Phosphorylated NFH in CSF was quantified by electrochemiluminescence immunoassay (Meso Scale Discovery).
- T-regulatory cell frequency was assessed by multicolour flow cytometry (CD4⁺CD25⁺FOXP3⁺ subset, expressed as % of CD4⁺ cells).
- CCL2 plasma concentration was measured using multiplex bead-based immunoassay (Bio-Plex, Bio-Rad).

All variables were checked for outliers; values > ± 3 SD were winsorised. Non-normally distributed biomarkers (NFH, CCL2) were analysed on their natural scales and in log₁₀-transformed form in sensitivity analyses, yielding comparable results.

### TRICALS score computation

For each participant, the continuous TRICALS survival score was calculated using the published coefficients from the European Network for the Cure of ALS (ENCALS) model, incorporating baseline age, diagnostic delay, ALSFRS-R, and site of onset. The score was centred (mean = − 4.48, SD = 1.6 in this cohort) and entered as a continuous variable in Cox proportional-hazards models [4].

### Statistical analysis and modelling framework

We used Cox proportional-hazards regression to address two prespecified scientific questions:

#### Analysis 1: substitution analysis

Can a clinical phenotype model replace a molecular biomarker in survival modelling?

To address this, we reproduced the published full biomarker survival model from MIROCALS, but substituted CSF pNFH with the TRICALS risk score. This model included:

- ALSFRS-R
- Age
- Regulatory T-cell frequency
- Plasma CCL2
- TRICALS risk score (in place of pNFH)
- Treatment arm
- Interaction of TRICALS risk score with treatment arm

Model performance was compared with the published biomarker model using likelihood ratio χ^2^ statistics and − 2 log likelihood (− 2LL).

#### Analysis 2: augmentation analysis

Does clinical phenotype add independent information beyond molecular biomarkers?

To address this, we fitted the published full phenotype and biomarker model and then added the TRICALS risk score as an additional covariate. We further tested an interaction between TRICALS risk score and treatment arm as a sensitivity analysis.

The published full biomarker model included:

- ALSFRS-R
- Age
- Regulatory T-cell frequency
- Plasma CCL2
- CSF pNFH
- Treatment arm
- CSF pNFH × treatment interaction

Improvements in model fit after addition of TRICALS risk score were assessed using likelihood ratio tests (Δχ^2^, Δdf).

### Model estimation and inference

Hazard ratios (HRs) and 95% confidence intervals were derived from exponentiated regression coefficients. Model fit was assessed using − 2 log likelihood (− 2LL), overall model χ^2^ and likelihood ratio tests for nested comparisons. Covariate means were extracted to support interpretation of interaction terms. All analyses were performed stepwise, matching the analytical approach of the original MIROCALS publication. SPSS v29 was used for analysis [7].

### Missing data handling

The analysis followed a complete-case approach, consistent with the original trial’s statistical plan. No imputation was performed.

### Assessment of model assumptions

Proportional hazards assumptions were evaluated using log–log survival plots and Schoenfeld-type residual inspection. pNFH was retained in its original measurement units to ensure consistency with the original trial analysis; no scaling or transformation was applied. Linearity of continuous covariates was verified through visual inspection and comparison of continuous versus categorised forms.

### Ethics

The study was conducted in compliance with Good Clinical Practice (ICH guidelines) and in accordance with the ethical principles of the Declaration of Helsinki, with approvals from the relevant Institutional Ethical Review Bodies in the UK (Integrated Research Application System, #207,544) and France (Comité de Protection des Personnes Ile de France-VI, #37–16). An independent Data Safety Monitoring Board (DSMB) reviewed the unblinded data for safety only at agreed stages of the study. The MIROCALS study sponsor was the Centre Hospitalier-Universitaire de Nîmes (CHU-Nîmes), France. Our analyses were conducted on deidentified data, in accordance with the informed consent provided by trial participants and the original ethical approvals governing MIROCALS. The original trial received full ethical approval at all participating centres and all participants provided written informed consent. The present analysis used fully anonymised data provided under the trial’s data-sharing agreement. MIROCALS is registered with ClinicalTrials.gov (NCT03039673).

## Results

### Participant characteristics

220 randomised participants had complete baseline data for all covariates included in the multivariable analyses as described in the original study report. Among these, 90 participants reached the survival endpoint and 130 were censored. The baseline demographic, clinical, and biomarker variables were broadly comparable across treatment arms. TRICALS scores were normally distributed without evidence of outliers or group imbalance, with a mean of − 4.47 and SD 1.3.

### Substitution of CSF pNFH with TRICALS risk score in the full biomarker model

To test whether clinical phenotype could substitute for molecular biomarkers, the published MIROCALS biomarker model was refitted with CSF pNFH replaced by the TRICALS risk score and a TRICALS score × treatment interaction. Under this specification, overall model performance was substantially reduced compared with the published benchmark (− 2LL 928.25 vs 793.36). The TRICALS score × treatment interaction was statistically significant (HR 0.72; 95% CI 0.54–0.96; *p* = 0.025), indicating that baseline clinical phenotype captured treatment-response heterogeneity to a measurable extent and that a treatment effect would still have been detected. However, the improvement in model fit attributable to the TRICALS score × treatment interaction was modest (Δχ^2^ = 4.76) and markedly smaller than that achieved by the CSF pNFH × treatment interaction in the published model (Δχ^2^ = 14.24).

### Correlation between TRICALS risk score and CSF pNFH

**Fig. 1:**
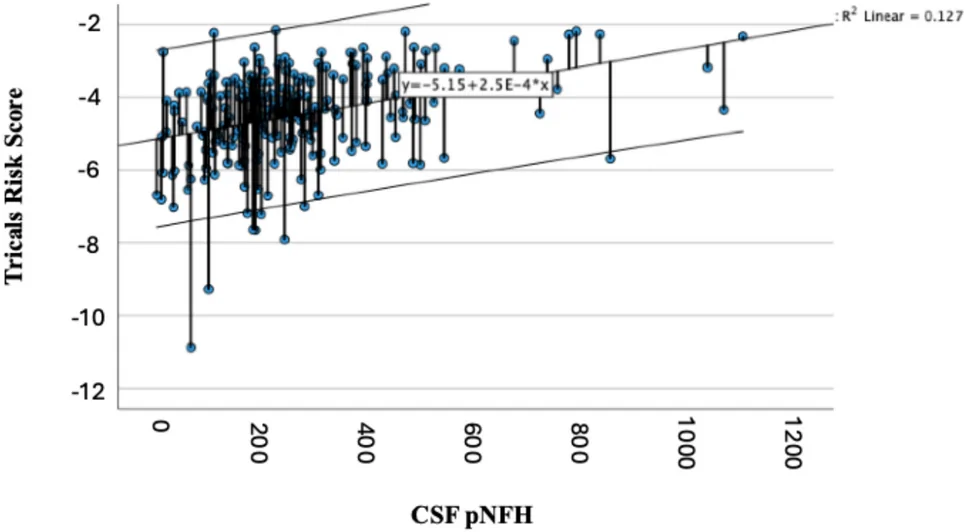
Scatter plot showing the relationship between baseline TRICALS risk score and phosphorylated neurofilament heavy chain (CSF pNFH) concentration in 220 participants from the MIROCALS trial. Each point represents an individual participant. The solid line represents the fitted linear regression. A statistically significant positive correlation was observed (Pearson *r* = 0.357; *p* < 0.001). The coefficient of determination (*R*^2^ = 0.127) indicates that 12.7% of the variance in CSF pNFH is explained by the TRICALS risk score

TRICALS risk score was moderately correlated with CSF pNFH concentration (Pearson *r* = 0.357; *p* < 0.001; *n* = 220). The coefficient of determination (*r*^2^ = 0.127) indicates that TRICALS score accounted for approximately 12.7% of the variance in CSF pNFH (Fig. 1).

### Addition of pNFH and pNFH × treatment interaction

Incorporation of the CSF pNFH and pNFH × treatment interaction produced a further significant improvement in fit (− 2LL 869.62; χ^2^ = 72.84, df = 4; *p* < 0.001), with an incremental gain of Δχ^2^ = 14.24 (*p* < 0.001) over the additive model. The interaction term was highly significant (Wald χ^2^ = 14.97; *p* < 0.001). With inclusion of this interaction, treatment became a significant predictor of outcome (HR 0.28; 95% CI 0.12–0.63; *p* = 0.002). TRICALS score (HR 1.24; *p* = 0.032) and CSF pNFH (*p* = 0.002) remained independently associated with survival.

This model therefore simultaneously captured:

- Baseline clinical severity (TRICALS risk score),
- Biological disease activity (pNFH), and
- Treatment–response heterogeneity (pNFH × treatment and TRICALS score × treatment).

### Model summary and comparison with the published biomarker model

The published MIROCALS biomarker model, comprising ALSFRS-R, age, regulatory T-cell frequency, plasma CCL2, CSF pNFH, treatment arm, and the pNFH × treatment interaction, achieved the strongest overall fit (− 2LL 793.36).

Substitution of CSF pNFH with TRICALS risk score resulted in reduced model performance (− 2LL 928.25), despite a statistically significant TRICALS × treatment interaction (HR 0.72, 95% CI 0.54–0.96; *p* = 0.025; Δχ^2^ = 4.76).

In contrast, incorporation of both CSF pNFH and the pNFH × treatment interaction and TRICALS score-based models produced substantial improvements in fit and identified a treatment effect.

## Discussion

This reanalysis of the MIROCALS randomised trial data demonstrates that clinical phenotype and molecular biomarkers in amyotrophic lateral sclerosis capture overlapping but non-equivalent biological information and should not be treated as interchangeable in survival modelling or trial stratification. Specifically, the TRICALS risk score does not replace CSF phosphorylated neurofilament heavy chain (pNFH) as a predictor of treatment-response heterogeneity, but instead provides complementary information when incorporated into a biomarker-based framework.

In the substitution analysis, replacing pNFH with the TRICALS risk score within the published biomarker model resulted in a deterioration in model performance. Although the TRICALS score is strongly associated with survival, and its interaction with treatment reached statistical significance, the improvement in fit was modest and substantially weaker than that obtained with the pNFH × treatment interaction. This finding indicates that clinical phenotype alone does not capture the biological processes underlying differential response to IL-2_LD_ and cannot be used as a surrogate for neuroaxonal injury markers in this context.

In contrast, the augmentation analysis demonstrated that adding pNFH to the TRICALS score phenotype-based model produced a large and statistically robust improvement in prognostic performance. Neurofilament burden emerged as a dominant independent predictor of survival, consistent with extensive evidence linking neurofilament concentrations to the rate and extent of neuroaxonal degeneration in ALS. When the pNFH × treatment interaction was introduced into the TRICALS score-based model, the interaction produced a further significant improvement in model fit and restored a statistically robust treatment effect, closely matching the magnitude observed in the published trial analysis.

This study has limitations. It is a post-hoc analysis of a published clinical trial, and is not therefore intended to be a formal reanalysis of the trial data, but rather, a way to test the hypothesis that a phenotypic score, in the form of the TRICALS risk score, might be able to replace or substitute for a fluid biomarker of neurodegeneration, in the form of CSF pNFH.

The findings have direct implications for ALS trial design. First, they argue against the use of clinical risk scores as substitutes for biological markers in treatment-stratified analyses. Although composite phenotype scores are valuable for prognostic adjustment, they do not capture the molecular processes that determine therapeutic response. Second, they support the integration of disease-activity biomarkers into trial modelling frameworks, particularly when evaluating biologically targeted interventions [8].

More broadly, this study illustrates the need to develop treatment-specific biomarker frameworks in neurodegenerative disease trials. As therapeutic strategies increasingly target distinct biological pathways, immune modulation, protein aggregation, RNA metabolism, mitochondrial dysfunction, no single biomarker is likely to be universally informative. Instead, optimal trial design will require the identification of molecular markers that directly index the biological processes targeted by the intervention [9, 10].

Taken together, these results support a pragmatic precision-medicine strategy for ALS trials. Clinical phenotype should be used for baseline risk adjustment, but not as a surrogate for molecular stratification. Disease-activity biomarkers, particularly neurofilament measures such as CSF pNFH, are essential for identifying treatment-responsive subgroups. Integrating both within a unified modelling framework provides mechanistic insight, statistical efficiency, and a practical route towards more interpretable and therapeutically informative ALS clinical trials.

## Supplementary Information

Below is the link to the electronic supplementary material.

Supplementary file1 (PDF 45 KB)

## Data Availability

The MIROCALS study data are subject to third-party access restrictions. The authors did not generate these data and are not authorised to share individual-level participant data directly. Access to MIROCALS de-identified data may be requested (from 12 months after the original study publication) by contacting the MIROCALS study team at drc@chu-nimes.fr, specifying the variables requested and the intended use including study objectives and research plan. The MIROCALS study protocol and data dictionary are also available via this contact.
