# Supplementary material for: TRICALS risk score augments but cannot replace neurofilament as a prognostic biomarker in ALS
Source: J Neurol. 2026 Oct 6;273(10):643. doi: 10.1007/s00415-026-14040-4 (PMC13642438; doi:10.1007/s00415-026-14040-4)
Supplement: Supplementary file 1 [file 415_2026_14040_MOESM1_ESM.pdf]

Title: TRICALS risk score augments but cannot replace neurofilament as a prognostic biomarker in ALS

Ahmad Al Khleifat[1, 2, 3*], Andrea Malaspina [4, 5], Janine Kirby[6], Timothy Tree[7], Pamela J Shaw[6, 8], P. Nigel Leigh[9], Ammar Al-Chalabi[1, 10 *]

Author Affiliations:

1.Department of Basic and Clinical Neuroscience, Institute of Psychiatry, Psychology and Neuroscience, King's College London, London SE5 8AF, UK

2.School of Medicine, The University of Jordan. Amman, Jordan.
3. Perron Institute for Neurological and Translational Science, Nedlands, Australia

4.UCL Queen Square MND Care and Research Centre, Institute of Neurology, University College London, London, UK

5.Centre of Neuroscience and Trauma, Blizard Institute, Queen Mary University London, London, UK

6. Sheffield Institute for Translational Neuroscience, School of Medicine and Population Health, University of Sheffield, Sheffield, UK

7.Department of Immunobiology, Faculty of Life Sciences and Medicine, King's College London, London, UK

8.NIHR Sheffield Biomedical Research Centre, University of Sheffield and Sheffield Teaching Hospitals NHS Foundation Trust, Sheffield, UK

9. Department of Neuroscience, Brighton and Sussex Medical School, Falmer, Brighton, BN1 9PX, UK

10. Department of Clinical Neurosciences, King's College Hospital, London SE5 9RS, UK

Correspondence to: Ahmad Al Khleifat

Department of Basic and Clinical Neuroscience, Institute of Psychiatry, Psychology and Neuroscience, King's College London, 16 De Crespigny Park, London SE5 8AF, UK

Email: ahmad.al_khleifat@kcl.ac.uk

and Ammar Al-Chalabi

Department of Basic and Clinical Neuroscience, Institute of Psychiatry, Psychology and Neuroscience, King's College London, 16 De Crespigny Park, London SE5 8AF, UK

Email: ammar.al-chalabi@kcl.ac.uk

Keywords: amyotrophic lateral sclerosis; ALS; Biomarker; Clinical Trials

Appendix

MIROCALS Study Group

Gilbert Bensimon, P Nigel Leigh, Timothy Tree, Andrea Malaspina, Christine AM Payan, Hang-Phuong Pham, Pieter Klaassen, Pamela J Shaw, Ahmad Al Khleifat, Maria D M Amador, Shahram Attarian, Simon M Bell, Stéphane Beltran, Emilien Bernard, William Camu, Philippe Corcia, Jean-Christophe Corvol, Philippe Couratier, Véronique Danel, Rabab Debs, Claude Desnuelle, Aikaterini Dimitriou, John Ealing, Florence Esselin, Marie-Céline Fleury, George H Gorrie, Aude-Marie Grapperon, Adèle Hesters, Raul Juntas-Morales, Ivan Kolev, Géraldine Lautrette, Nadine Le Forestier, Christopher J McDermott, Nicolas Pageot, François Salachas, Nikhil Sharma, Marie-Hélène Soriani, Jemeen Sreedharan, Juliette Svahn, Nick Verber, Annie Verschueren, Ozlem Yildiz, Carey M Suehs, Safaa Saker-Delye, Claudie Muller, Christophe Masseguin, Hana Hajduchova, Janine Kirby, Cecilia Garlanda, Massimo Locati, Henrik Zetterberg, Bernard Asselain, Ammar Al-Chalabi.

Coordination: Fatima Ben Lahcen, Sareema Javaid, and Elodie Jouin. Clinic: Raquel Nsue Sha Akieme, Sébastien Alphandéry, Matthew Appleby, Salah Bakkouche*, Carole Barré, Catherine Bellot, Bethan Blackledge, Geetha Boyapati, Camille Brodziack, Judith Brooke, Julie Catteau, Alizé Chalançon, Theresa Chiwera, Chukwuma Chukwu, Paul-Foster Cofie, Arnaud Declemy, Mary Flowerdew, John Goodfellow, Daniel Hansen, Nacime Heddadji, Steven Henderson, Mbombe Kazoka, Hafida Kribich, Atika Kuhn, Clémence Labetoulle, Marie-France Le Labousse, Praveen Macherla, Keith Mayl, Hélène Merle, Emanuela Molinari, Julie Moutarde, Mohammed Mouzouri, Dee Mullan, Camille Neuillet, Lynn O’Donohoe, Marie Pleuvret, Valérie Santraine, Juliet Staunton, Maëlle Vomscheid, and Stacy Young. Laboratory process: Bertrand Accart, Hélène Blasco, Abigail Brown, Clément Bruno, Alex Daniel, John De Vos, David Devos, Iain Dow, Nathalie Dufay, Guillaume Fiard, Sylvie Forlani, Florence Glaudet, Ludmila Jornea, Béatrice Leloup-Poilane, Vittoria Lombardi, Nathan Mairs, Guillaume Olombel, Julie Oxton, Fabiola Puentes, Clare Rizzo-Singh, Aleksey Shatunov, Lana Taylor, Jean-Luc Veyrune, and Dominika Wlazly. Site pharmacy: Guillaume Becker, Florianne Bel, Christelle Boczek, Alexandre Bolle, Helen Bowler, Carol Carter (nee Jaques), Stuart Chandler, Fanny Charbonnier-Beaupel, Florence Collado, Bénédicte Deluca, Joanna Flanagan, Caroline Gervaise, Helen Hamilton, Jason Howard, Claire Keatley, Nathalie Kerneur, Gaynor Notcheva, Elodie Péguet, Françoise Renon-Carron, Isabelle Roch-Torreilles, Colin Rodden, Tenesa Sargent, and Emmanuelle Schreiber.
